# The impact of peer and media pressure on muscle dysmorphia for men and women in the general German population is mediated by the internalization of muscularity ideals

**DOI:** 10.1186/s40337-026-01718-3

**Published:** 2026-08-04

**Authors:** Marietta Lieb, Christopher Zaiser, Nora M. Laskowski, Georg Halbeisen, Georgios Paslakis

**Affiliations:** 1 Department for Psychosomatic Medicine and Psychotherapy, Ruhr-University Bochum, LWL- University Hospital, Alexandrinenstraße 1-3, 44791 Bochum, Germany; 2 Faculty of Business Administration and Economics, OWL University of Applied Sciences and Arts, Lemgo, Germany

## Abstract

**Background:**

Muscle dysmorphia (MD) is characterized by an excessive concern of not being muscular enough. Sociocultural influences appear to play a central role in the internalization of muscular beauty ideals and the development of MD. No study has examined the potential mediating pathways from sociocultural pressure sources through internalized beauty standards to an algorithm-defined MD diagnosis in the general German population.

**Methods:**

A cross-sectional survey was conducted among adults (≥ 18 years) in Germany via the Online Platform Prolific^®^. Participants completed standardized questionnaires assessing sociocultural influences and internalization of different beauty standards (SATAQ-4R) and a clinically algorithm-defined MD, operationalized using criteria by Pope et al. [4], following the approach described by Mitchison et al. [14]. Data were weighted to approximate representativeness for the German population across the key demographics age and gender.

**Results:**

In the total sample, a binary logistic regression indicated that internalization of the beauty standards Thin/Low body fat, Muscularity, and General Attractiveness significantly predicted algorithm-defined MD, whereas sociocultural pressure sources such as Family, Peers, Significant Others, and Media were not significant. In linear regression models, Media pressures predicted all forms of internalization, while Peer pressure predicted only Muscularity internalization. Mediation analyses indicated that internalization, particularly internalization of ideals of Muscularity, significantly mediated the relationship between Media and Peer pressures and algorithm-defined MD. Gender-specific analyses also showed Muscularity internalization as the key mediator for both men and women. In men, internalization of General Attractiveness ideals additionally mediated the relationship between Media pressure and MD. In women, only Family had a significant effect on algorithm-defined MD, mediated via Muscularity Internalization.

**Discussion:**

Our findings highlight that internalization, particularly of the Muscularity ideal, appears to be a core mechanism linking sociocultural pressures to algorithm-defined MD. These results provide empirical evidence that interventions for MD need to focus on modifying internalized appearance ideals, especially the Muscularity ideal, to reduce MD symptoms in both men and women, with additional attention to General Attractiveness internalization in men.

**Supplementary Information:**

The online version contains supplementary material available at 10.1186/s40337-026-01718-3.

## Abstract 2

Muscle dysmorphia (MD) is a condition in which people experience strong concerns about not being muscular enough. This study investigated how social influences, such as pressure from the Media, Peers, Family, and Significant others, are related to MD in the German population. We examined whether these influences contribute to MD through the internalization of appearance ideals, meaning the extent to which people adopt certain body ideals as personal standards. Our findings showed that adopting the muscularity ideal as a personal standard was the most important factor related to MD in both men and women. Media and Peer pressures were linked to MD mainly through the adoption of this appearance ideal. In men, the adoption of general attractiveness ideals played an additional role. In women, Family pressure uniquely contributed to MD through the muscularity ideal, highlighting gender-specific pathways. These findings suggest that prevention and treatment approaches for MD should focus on addressing the adoption of these appearance standards, particularly the muscularity ideal.

**Supplementary Information:**

The online version contains supplementary material available at 10.1186/s40337-026-01718-3.

## 1. Background

In a society where the ideal body is increasingly defined by muscularity and leanness [1, 2], the pursuit of a “perfect” muscular physique may, for some individuals, become pathological. This phenomenon is known as muscle dysmorphia (MD). According to the DSM-5 [3], MD is a subtype of body dysmorphic disorder (BDD), characterized by an excessive preoccupation with perceived flaws in physical appearance, repetitive behaviors (e.g., mirror checking) or mental preoccupation (e.g., comparing oneself to others). It causes significant distress or impairment and is not better explained by concerns about body fat or weight, thus excluding individuals with an eating disorder. The specifier ‘with muscle dysmorphia’ applies when the main concern is being too small or insufficiently muscular.

Pope et al. [4] proposed more specific criteria that mirror the DSM-5 but include additional details to better capture the clinical features of MD and its distinct focus on muscularity. These criteria include -next to the belief that one’s body is insufficiently lean and muscular- behaviors such as excessive weightlifting and strict dieting, a disruption of daily routines due to compulsive exercise or dieting, continuation of these behaviors despite awareness of the negative consequences, clinically significant distress, and impairments in social, occupational, and recreational functioning (e.g., avoidance of situations where the body is exposed).

Considering the importance of excessive training in MD, it is not surprising that this disorder is most commonly observed in samples of weightlifters and bodybuilders, with estimated prevalence rates ranging from roughly 17 to 58.3% across studies [5–10]. However, when broader population samples are examined, prevalence estimates appear considerably lower. Applying the Muscle Dysmorphic Disorder Inventory (MDDI [11]), a self-report questionnaire assessing MD symptoms, 25.7% of men, 11.2% of women, and 18.0% of transgender individuals were identified to be “at risk” for MD in a Canadian study [12]. Similar rates are observed among German “fitness enthusiasts”, with 25% of men and 16% of women [13]. However, the MDDI only identifies “at risk” populations and does not provide a clinical diagnosis. In contrast, when MD is operationalized in line with diagnostic criteria proposed by Pope et al. [4], it more closely approximates a clinical diagnosis, yielding substantially lower prevalence estimates of 2.2−2.8% among adolescent boys and men and 1.4% among girls [14, 15]. Overall, these findings suggest that MD is more common in men than in women.

Besides gender, various factors have been associated with MD, such as anxiety and depression, neuroticism, negative affect, perfectionism, traumatic experiences as well as negative self-concept and self-esteem [5, 7, 16–18]. There is also an increasing focus on the impact of sociocultural influences and contemporary societal beauty standards on the manifestation of MD. In Western culture, the contemporary male body ideal emphasizes muscularity and a lean, athletic physique [1]. Although the female body ideal has often been associated with thinness and low body fat [19], it also increasingly incorporates elements of muscularity, favoring a toned and defined figure that combines both thinness and muscularity [1, 2, 20–23]. Emerging evidence suggests that social environments and especially media influences may contribute to the internalization of these ideals (i.e., the process of adopting societal standards as personal beliefs and goals), resulting in increased body dissatisfaction, particularly concerning muscularity and body fat [24–29].

Psychosocial pressures and the internalization of sociocultural standards can be conceptualized within the Tripartite Influence Model (TIM). First introduced by Thompson et al. [19], it explained the development of eating disorders and related body image disturbances in women and girls. It posits three primary sources of influence -Peers, Parents, and Media- contributing to the emergence of body dissatisfaction and disordered eating via the internalization of societal appearance ideals and higher tendencies for comparisons in appearance. Keery et al. [30] later provided empirical validation, confirming the model’s predictive pathways. The model was subsequently refined by Tylka et al. [27], emphasizing the role of internalizing societal appearance ideals as the central mechanism rather than focusing solely on social comparison and adding romantic partners as another source of sociocultural pressure. Lately, the TIM has increasingly been applied to men and the development of dissatisfaction with muscularity and muscularity enhancement behaviors, suggesting that similar social and mediating factors might be involved in MD as in eating disorders [31].

Several studies underscore the relevance of sociocultural influences and internalized beauty ideals in muscle building behavior and MD as proposed by the TIM. Regarding media influences, higher social media use and greater overall screen time have been linked to higher MDDI scores, while exposure to fitness related contents (e.g., fitspiration) has been associated to muscularity dissatisfaction [26, 32, 33]. More generally, perceived media pressure has been associated with MD (measured using the Muscle Dysmorphia Questionnaire, MDQ) [34] and has been identified as the strongest predictor for overall drive for muscularity [35]. Beyond media effects, peer pressure also appears to play an important role in the development of MD, as it has been associated with both scores on the MDQ [34] and with “probable” clinical MD (based on Pope’s criteria [4]) [36]. Internalization of beauty standards further seem to play a role in MD. Greater internalization of muscularity ideals has been associated with higher MD symptomatology, including MDDI scores and “probable” MD in male samples [36, 37]. Importantly, the relationship differs depending on the type of internalized ideal: internalization of muscularity and overall attractiveness are positively associated with MD symptoms, whereas the endorsement of the Thin/Low Body Fat ideal is associated with a lower likelihood of such symptoms [36]. In addition, there is evidence for an interaction effect, indicating that muscularity internalization is more strongly associated with MD symptoms when thinness internalization is low [37]. Overall, these studies support the idea that the internalization of ideals represents a key mechanism linking sociocultural exposure to MD symptomatology.

Fewer studies have gone a step further to model the pathways through which sociocultural pressure translates into MD. For instance, work in North American samples [16, 28] has applied the Dual Pathway Model [38], which emphasizes the role of negative affect but does not distinguish between different sources of sociocultural pressure. Empirical findings indicate that social pressure is related both directly to drive for muscularity and indirectly via internalization of the muscularity ideal in men. Drive for muscularity, in turn, has been linked to MD symptoms (MDDI scores), both directly and indirectly via muscle-enhancing behaviors and negative affect, although the latter pathway was only evident among men reporting higher levels of narcissistic vulnerability [16]. At the same time, sociocultural appearance pressures (total sum score of the Sociocultural Attitudes Toward Appearance Questionnaire 3, SATAQ-3) have been linked to MD symptoms via body dissatisfaction, whereas negative affect did not mediate this relationship [28]. Taken together, these models converge on the view that sociocultural pressure influences MD largely indirectly -via internalization, drive for muscularity, or body dissatisfaction.

Although some studies have examined associations between sociocultural influences, internalization of beauty standards, and MD or muscularity-related behaviors and symptoms, several gaps remain. No study to date has simultaneously investigated the pathways from distinct sociocultural pressure sources (Family, Peers, Significant Others, and Media) through the internalization of Thinness, Muscularity, and General Attractiveness ideals to a clinically approximated MD diagnosis [4] in a general German sample including both men and women. Most prior research has focused exclusively on men and has relied on dimensional screening tools for MD. Our study therefore aims to provide a novel contribution by addressing all of these aspects concurrently and by additionally examining gender-specific pathways.

## 2. Methods

### 2.1. Setting and procedure

This study employed a cross-sectional online survey design. Participants were recruited via the established online research platform Prolific.com. After providing informed consent, participants completed a set of standardized online questionnaires. Participants received a compensation of £7 (approximately €8.18) for taking part in this study. Inclusion criteria were age ≥ 18 years, voluntary participation, full legal capacity and sufficient German language proficiency, which were confirmed by participants on an initial entry page by means of an active consent button. The study was reviewed and approved by the Ethics Committee of the Faculty of Medicine at Ruhr-University Bochum, East Westphalia Campus, and was conducted in accordance with the Declaration of Helsinki. The study was preregistered on AsPredicted (No. 235974).

### 2.2. Measures

#### The sociocultural attitudes toward appearance questionnaire-4 revised

The Sociocultural Attitudes Toward Appearance Questionnaire-4 Revised (SATAQ-4R) [39] is an internationally recognized instrument assessing both the internalization of societal beauty ideals and the perceived pressure to conform to these ideals. The German SATAQ-4R [40] mirrors the English original and exists in two gender-specific versions, with 28 items for men and 31 items for women. Each version includes seven subscales in total: three measuring the extent of internalization of Thinness, Muscularity, and General Attractiveness ideals, and four assessing perceived pressure from Family, Peers, Significant Others, and Media. All items are rated on a 5-point Likert scale, ranging from “Definitely Disagree” to “Definitely Agree.”

#### Operationalization of algorithm-defined muscle dysmorphia

To obtain a clinical classification of MD, we applied the criteria proposed by Pope et al. [4] and operationalized them following the approach described by Mitchison et al. [14]. For this purpose, specific subscales and items of the Muscle Dysmorphic Disorder Inventory (MDDI) [11, 13], the Drive for Muscularity Scale (DMS) [41, 42], the Eating Disorder Examination-Questionnaire (EDE-Q) [43, 44] and the Kessler Psychological Distress Scale (K-10) [45, 46] were used. The operationalized scores from these instruments were subsequently integrated into a diagnostic algorithm to classify participants with algorithm-defined MD. Detailed information on the operationalization is provided in Supplementary Table S1, and descriptions of all applied instruments are available in Supplementary Table S2.

### 2.3. Statistics

#### Weighting

To ensure that the results reflect the demographic structure of the German population, the data were weighted using post-stratification (raking) weights for key demographic variables such as age and gender, based on official population benchmarks. All analyses in this study were conducted using the weighted data, enhancing the validity and generalizability of the findings.

#### Statistical analysis

Data were processed and analyzed using R (version 4.4.1 and 4.5.3) [47], primarily with the package survey [48]. Other packages used included dplyr [49], psych [50], Hmisc [51], and DescTools [52]. All analyses were conducted using the svyglm functions from the survey package to account for the survey design, the weighted sample, and to obtain appropriate standard errors, confidence intervals, and statistical inference. We depicted descriptive statistics including frequencies, means (Ms), medians (MDs), standard deviations (SDs), and ranges. Weighted prevalence estimates for algorithm-defined MD and their 95% confidence intervals were calculated based on the gender-specific weighted cutoff [53]. Group differences were assessed using independent t-tests. When assumptions of parametric testing were violated, Welch’s t-tests or Mann-Whitney U tests were applied. Effect sizes were calculated using both Cohen’s d and r and interpreted according to conventional thresholds: for Cohen’s d, small (0.2 ≤ d < 0.5), medium (0.5 ≤ d < 0.8), and large (d ≥ 0.8); for r, small (0.1 ≤ *r* <0.3), medium (0.3 ≤ *r* <0.5), and large (*r* ≥0.5). Associations between categorical variables were analyzed using Chi^2^-tests, with effect sizes reported as Phi coefficients and interpreted as follows: small (0.10 ≤ φ < 0.30), medium (0.30 ≤ φ < 0.50), and large (φ ≥ 0.50). For correlational analyses, Pearson’s correlation coefficient was used. To examine associations between study variables, we estimated a series of weighted linear and binary logistic regression models for the total sample as well as separately for men and women. In all regression analyses and the overall path model, age, gender, and BMI were included as control variables. These regression models jointly constituted the regression-based path model, with sociocultural influence variables (Family, Peers, Significant Others, and Media) as predictors, internalization variables (Thin/Low Body Fat, Muscularity, and General Attractiveness) as mediators, and algorithm-defined MD as the binary outcome. Indirect effects were estimated as the product of the respective a- and b-paths and should be interpreted as exploratory, given the binary outcome and the observational study design. A significance level of *p* <0.05 was applied to all statistical tests.

## 3. Results

A total of *N* = 1445 men and women participated in the study. Additionally, 23 participants reporting “other” gender identity or not providing information on their gender were included in the original unweighted sample but were excluded from further analyses due to the small subgroup size. After weighting, the sample consisted of 704 (48.7%) men and 741 (51.3%) women. When applying the diagnostic algorithm, a clinically algorithm-defined MD was prevalent in 37 (2.6%) of all participants. For women, the prevalence of MD was lower than in men (12, 1.7% vs. 25, 3.5%). Weighted estimates are based on the male/female analytic sample using cutoffs derived from the weighted distribution. As a weighting diagnostic, Kish effective sample size for the weighted analytic sample was 151.1 overall (ESS/*N* =0.104; design effect = 9.59), with gender-specific ESS values of 93.9 for men and 64.0 for women. A design effect of this magnitude indicates that the weighting substantially reduced the effective information in the sample; the weighted prevalence estimates should therefore be interpreted with corresponding caution, and the unweighted estimates reported elsewhere [53] provide a more stable reference point.

### 3.1. Sample characteristics

The sociodemographic characteristics of the total weighted sample are displayed in Table 1. Women had a significantly lower BMI (*p*<0.001), lived significantly more often in single-person households (*p*<0.001), and more often reported being non-heterosexual (*p*<0.001) compared to men.

**Table 1:** Sociodemographic characteristics

Variable	Total	Men	Women	T-Test/Chi^2^-Test
Age	Mean (SD), median (range)			
	48.6 (16.7), 51 (18–75)	46.5 (15.2), 49 (18–71)	50.5 (17.8), 54 (18–75)	t(1443) = 1.316, *p*=.188, d = − 0.242
BMI	26.1 (5.6), 24.8 (14–54)	27.2 (5.0), 25.9 (16–54)	25.0 (5.9), 23.8 (14–53)	t(1442)=−2.831 *p* =.005, d=0.416
Marital status Married^1^ Not married	N (%)			
	568 (39.3) 877 (60.7)	325 (46.1) 379 (53.9)	243 (32.8) 498 (67.2)	χ^2^(1) = 26.99, *p*=0.11, φ = 0.137
Education < 12 years ≥ 12 years	240 (16.6) 1205 (83.4)	115 (16.4) 589 (83.6)	125 (16.9) 615 (83.1)	χ^2^(1) = 0.08, *p*=0.96, φ = 0.007
Persons in household 1 Person > 1 Person Missing	442 (30.6) 989 (68.4) 14 (1.0)	165 (23.4) 532 (75.5) 8 (1.2)	277 (37.5) 457 (61.8) 6 (0.8)	χ^2^(1) = 33.67, *p*=0.04 φ = 0.153
Migration history^2^ Yes No	230 (15.9) 1215 (84.1)	124 (17.5) 581 (82.5)	106 (14.4) 634 (85.6)	χ^2^(1) = 2.71, *p*=0.34, φ = 0.043
Sexual Orientation Heterosexual Non-heterosexual^3^ Missing	1265 (87.5) 167 (11.6) 13 (0.9)	665 (94.4) 37 (5.3) 2 (0.3)	600 (81.0) 130 (17.6) 11 (1.4)	χ^2^(1) = 54.66, *p*<0.001 φ = 0.195

^1^married includes registered partnership, not married includes being single, divorced, or widowed, ^2^Migration history: a person has a migration background if they themselves or at least one parent was not born as a German citizen, ^3^Non-heterosexual includes homosexual, bisexual, and other

### 3.2. Group differences for SATAQ scales

Women showed significantly higher scores for General Attractiveness, Family, Media, and SATAQ Total, whereas men scored significantly higher on the Muscularity subscale (*p* <0.05). Among men, those with algorithm-defined MD reported significantly higher scores for Muscularity, General Attractiveness, Peers, Media, Internalization Total, Pressure Total, and SATAQ Total (*p* <0.05) compared to men without MD. Women with algorithm-defined MD exhibited significantly higher scores for Thin/Low Body Fat, Muscularity, General Attractiveness, Peers, Significant Others, Internalization Total, Pressure Total, and SATAQ Total (*p* <0.05) compared to women without MD. Table 2 presents the mean scores of the SATAQ scales separately for men and women, as well as for men and women with and without algorithm-defined MD.

**Table 2:** Mean Scores on SATAQ subscales by gender and MD

	Total Men	Total Women	Men without MD	Men with MD	Women without MD	Women With MD
	M (SD)					
SATAQ total	2.05 (0.65)*^b^	2.41 (0.63)*^b^	2.03 (0.64)*^c^	2.63 (0.51)*^c^	2.4 (0.63)*^d^	3.02 (0.36)*^d^
Internalization						
Thin/low body fat Muscularity General attractiveness Total	2.65 (0.99) 2.88 (1.05)*^a^ 3.32 (1.02)*^a^ 2.95 (0.76)	2.81 (1.04) 2.52 (0.91)*^a^ 3.69 (0.80)*^a^ 3.01 (0.73)	2.65 (0.99) 2.82 (1.03)*^c^ 3.23 (1.01)*^c^ 2.92 (0.75)*^c^	2.70 (1.13) 4.23 (0.73)*^c^ 4.34 (0.65)*^c^ 3.76 (0.61)*^c^	2.81 (1.04)* 2.50 (0.90)*^e^ 3.68 (0.80)*^d^ 3.00 (0.73)*^d^	3.07 (0.63)* 3.82 (0.40)*^e^ 4.29 (0.48)*^d^ 3.73 (0.33)*^d^
Pressure						
Family Peers Significant others Media Total	1.56 (0.70)*^a^ 1.73 (0.85) 1.49 (0.76) 1.95 (1.07)*^a^ 1.69 (0.69)	1.80 (0.96)*^a^ 1.58 (0.81) 1.44 (0.81) 2.39 (1.24)*^a^ 1.80 (0.80)	1.58 (0.70) 1.71 (0.83)*^b^ 1.48 (0.75) 1.91 (1.05)*^b^ 1.67 (0.69)*^b^	1.48 (0.63) 2.28 (1.09)*^b^ 1.71 (0.84) 2.92 (1.25)*^b^ 2.10 (0.66)*^b^	1.79 (0.96) 1.57 (0.81)*^d^ 1.43 (0.81)* 2.39 (1.25) 1.79 (0.80)*^d^	2.37 (0.99) 2.33 (0.73)*^d^ 1.96 (0.74)* 2.50 (0.95) 2.29 (0.57)*^d^

*Results differed significantly on a *p*<0.05 level

Effect size Cohen’s d was categorized as: a= small (0.2 ≤ d < 0.5), b= medium (0.5 ≤ d < 0.8), and c= large (d ≥ 0.8); Effect size r was categorized as: d = small (0.1 ≤ *r* <0.3), e = medium (0.3 ≤ *r* <0.5), f = large (*r* ≥0.5)

### 3.3. Preliminary path analyses

We conducted several preliminary analyses before testing the proposed mediation model. Preliminary correlational analyses between all SATAQ scales can be found in Supplementary Table S3a–c. When examining the direct effects of the SATAQ scales on the odds of an algorithm-defined MD diagnosis, controlling for BMI, gender, and age (X→Y), only BMI and the internalization variables (Thin/Low body fat, Muscularity, and General Attractiveness) were significantly associated with algorithm-defined MD, while the sociocultural pressure sources (Family, Peers, Significant Others, and Media) showed no direct effects. Among all variables, Muscularity internalization showed the strongest association with MD (see Table 3 for details).

**Table 3:** Binary logistic regression model: SATAQ scales as predictors of algorithm-defined MD (X→Y)

DV: MD	B	SE	OR (Exp [B])	95% CI for OR	Sig.
Age	−0.024	0.019	0.976	0.941–1.012	0.193
Gender	−0.651	0.763	0.521	0.117–2.327	0.394
BMI	−0.183	0.064	0.833	0.734–0.945	0.004*
Thin/low body fat	−0.371	0.178	0.690	0.487–0.978	0.037*
Muscularity	1.435	0.252	4.199	2.56–6.886	< 0.001*
General attractiveness	0.626	0.207	1.870	1.246–2.806	0.003*
Family	0.261	0.268	1.298	0.768–2.193	0.330
Peers	0.254	0.316	1.290	0.695–2.395	0.420
Significant others	−0.268	0.241	0.765	0.477–1.225	0.265
Media	0.070	0.233	1.073	0.68–1.694	0.763

*Results differed significantly on a *p*<0.05 level

When examining the effects of sociocultural pressure sources (Family, Peers, Significant Others, Media) on the internalization variables (Thin/Low Body Fat, Muscularity, General Attractiveness) (X→M), we found BMI and Media to be significantly associated with Thin/Low Body fat internalization. BMI, Media, Peers, and Gender were significantly associated with Muscularity ideals, with women reporting lower scores. BMI and Media were further significantly associated with General Attractiveness (see Table 4 details). All gender-specific analyses can be found in the Supplementary Tables S4a–f and the Supplementary Figures S1–2.

**Table 4:** Linear regression models: effects of sociocultural pressure sources on internalization (X→M)

	B	SE	95% CI for B	Sig.
DV: Thin/low body fat				
Age	−0.004	0.005	−0.014 to 0.005	0.375
Gender	−0.019	0.150	−0.312 to 0.274	0.898
BMI	−0.028	0.011	−0.050 to −0.006	0.013*
Family	0.106	0.078	−0.047 to 0.259	0.176
Peers	0.050	0.087	−0.120 to 0.220	0.563
Significant others	0.123	0.089	−0.052 to 0.297	0.168
Media	0.284	0.048	0.191 to 0.378	< 0.001*
DV: Muscularity				
Age	−0.002	0.004	−0.010 to 0.007	0.669
Gender	−0.496	0.133	−0.758 to −0.235	< 0.001*
BMI	−0.054	0.011	−0.075 to −0.033	< 0.001*
Family	0.081	0.067	−0.049 to 0.212	0.223
Peers	0.287	0.077	0.137 to 0.438	< 0.001*
Significant others	−0.070	0.081	−0.230 to 0.089	0.388
Media	0.112	0.047	0.020 to 0.204	0.017*
DV: General Attractiveness				
Age	−0.007	0.004	−0.015 to 0.002	0.122
Gender	0.266	0.137	−0.003 to 0.535	0.053
BMI	−0.027	0.012	−0.051 to −0.004	0.024*
Family	−0.011	0.075	−0.159 to 0.136	0.879
Peers	0.109	0.064	−0.016 to 0.234	0.088
Significant others	−0.004	0.089	−0.179 to 0.172	0.968
Media	0.183	0.045	0.095 to 0.271	< 0.001*

*Results differed significantly on a *p*<0.05 level

### 3.4. The mediation model

**Fig. 1:**
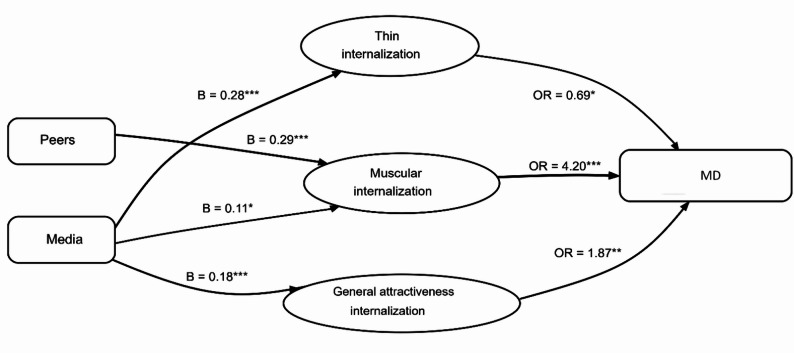
Significant indirect paths in the mediation model

To explore potential underlying mechanisms, we examined mediation pathways. Media was significantly associated with all three forms of internalization (Thin/Low body fat, Muscularity, and General Attractiveness), whereas the pressure source Peers was significantly associated only with the internalization of Muscularity ideals. In turn, all forms of internalization were significantly associated with MD diagnosis, with internalization of Muscularity showing the strongest association. These findings indicate that sociocultural pressure sources (Peers and Media) are indirectly related to algorithm-defined MD via internalization processes, particularly Muscularity internalization, which emerged as the central mediating mechanism. Figure 1 presents the mediation model with all significant indirect pathways. All indirect paths (X→M→Y), including non-significant paths, are presented in Table 5.

**Table 5:** Indirect paths of the mediation model (X→M→Y)

X→M→Y	Indirect effect (ab)	95% CI ab	Sig.
Family → Thin → MD	−0.039	−0.107 to 0.029	0.256
Peers → Thin → MD	−0.019	−0.084 to 0.047	0.577
SO → Thin → MD	−0.046	−0.123 to 0.032	0.250
Media → Thin → MD	−0.106	−0.212 to −0.002	0.049*
Family → Musc → MD	0.116	−0.075 to 0.308	0.234
Peers → Musc → MD	0.412	0.162 to 0.658	0.002*
SO → Musc → MD	−0.101	−0.332 to 0.131	0.393
Media → Musc → MD	0.161	0.018 to 0.302	0.027*
Family → GA → MD	−0.007	−0.100 to 0.085	0.879
Peers → GA → MD	0.068	−0.022 to 0.158	0.137
SO → GA → MD	−0.002	−0.112 to 0.107	0.968
Media → GA → MD	0.115	0.025 to 0.215	0.015*

BMI, age, and gender were included as control variables

*GA *General Attractiveness, *MD *algorithm-defined muscle dysmorphia, *SO *Significant Others, *Musc *Muscularity, *Thin *Thin/Low Body Fat

Gender-specific analyses identified significant indirect paths for Peers → Muscularity → MD (*p* =0.011), Media → Muscularity → MD (*p* =0.03), and Media → General Attractiveness → MD (*p* =0.048) in men, again highlighting Muscularity internalization as the central mediating mechanism, with an additional indirect effect via General Attractiveness for Media. In women, the only significant mediation was observed for Family → Muscularity → MD (*p*=0.024), also suggesting Muscularity internalization as the main mediating pathway.

## 4. Discussion

In this study, we examined the associations between sociocultural influences, internalization of beauty standards, and a clinically algorithm-defined MD diagnosis, as well as the mediating role of internalization of body-related ideals in the relationship between sociocultural influences and algorithm-defined MD in the general German population for both men and women.

We found a prevalence estimate of 2.6% of clinically algorithm-defined MD in the total sample, with a lower rate of MD in women (1.7%) than in men (3.5%). These rates are largely consistent with previous studies using the operationalization suggested by Mitchison et al., which reported prevalence estimates of 2.2–2.8% among adolescent boys and men and approximately 1.4% among girls [14, 15]. However, gender differences in prevalence rates should always be interpreted with caution, as eating- and body image–related disorders may manifest differently across genders, which may not be adequately captured by existing diagnostic criteria [54].

When examining the direct effects of all SATAQ scales on the odds of a algorithm-defined MD diagnosis in the total sample as preliminary analyses, only the internalization scales (Thin/Low Body Fat, Muscularity and General Attractiveness), along with BMI, were significantly associated with the outcome. Higher scores in Muscularity and General Attractiveness internalization, and lower scores in Thin/Low Body Fat internalization, were associated with increased odds for algorithm-defined MD. Ganson et al. [36] similarly found all forms of Internalization to be significantly associated with algorithm-defined MD, with the Thin/Low Body Fat ideal acting as a protective and the Muscularity and General Attractiveness ideals acting as risk factors. Higher internalization of Muscularity ideals has also been associated with higher MDDI scores [37]; however, contrary to our findings, that study [37] found higher rather than lower scores of thinness internalization to be linked to higher MDDI scores. Notably, that same study also reported an interaction in which muscularity internalization affected MDDI scores most strongly when thinness internalization was low to moderate, a pattern that partly converges with our finding, since both locate stronger MD-related effects at lower levels of thinness internalization, despite the opposing direction of the thinness main effect. In our study, lower Thinness internalization was linked to higher odds of algorithm-defined MD. This discrepancy may be explained by differences in outcome measurement, as we used a binary classification of algorithm-defined MD, while Klimek et al.’s study [37] employed the dimensional MDDI scale. An additional, non-exclusive explanation is that thinness internalization partly indexes eating disorder-relevant pathology, so differing degrees of unassessed subclinical eating disorder symptomatology across samples could contribute to the divergent direction of this association. Overall, most evidence suggests that higher Muscularity internalization is more closely linked to compulsive exercise and MD, whereas internalization of the Thin/Low Body Fat ideal appears to play a more protective role in MD-related outcomes. Notably, this same thinness-related construct -generally termed thin-ideal internalization in the eating disorder literature- has instead been more strongly and consistently associated with eating disorder symptoms [37, 55, 56]. Thin-ideal internalization may therefore carry opposite valence across the two conditions, protective for MD but a risk factor for eating disorders, while muscularity internalization stands out as the central driver of MD.

Regarding the direct effects of sociocultural pressure sources, we did not find any significant associations for Family, Peers, Significant Others, or Media on algorithm-defined MD when controlling for internalization variables and therefore accounting for potential overlaps in effects. However, when we examined indirect pathways in the mediation model, we found Peers to be a substantial influencing factor for MD, mediated via Muscularity Internalization. The influence of Peers on MD operated exclusively through Muscularity internalization as the central mechanism, with no significant effects for Thin/Low body fat or General attractiveness. This may reflect that muscularity is a particularly salient attribute in peer contexts. Also, You et al. [57] found that the impact of peers on body dissatisfaction was mediated via the Drive for Muscularity, but not the Drive for Thinness. Other studies also found Peer influence to be significantly associated with higher scores on the MDQ [34] as well as algorithm-defined MD [36], underpinning its role in muscularity-related outcomes.

We further found Media to be an important external source of pressure on MD, mediated via Thin/Low Body Fat, Muscularity, and General Attractiveness internalization. Media has also been linked to MD in previous studies. Total screen time has been associated with higher MDDI scores [26, 32] and Media pressure has been related to a stronger drive for muscularity [35] as well as to MD (as measured by the MDQ) [34], highlighting media as an important contributing factor. In contrast to peer pressure, the effect of media pressure was mediated through all forms of internalization, suggesting a broader and more pervasive influence than immediate peer interactions. It has also previously been considered as the most influential source regarding body dissatisfaction [58]. Nonetheless, the strongest mediation occurred through Muscularity. Another study examining mediation pathways similarly found that the total perceived sociocultural pressure from Parents, Friends, Significant Others, and Media (as measured by the Perceived Sociocultural Pressure Scale, PSPS) influenced the drive for muscularity through internalization of the Muscularity ideal, which in turn contributed to MD symptoms [16].

For Family and Significant Others, we did not find significant associations with internalizations, nor were there significant mediation pathways, suggesting that the primary influential sources for MD are centered on Peers and Media. This is consistent with previous research, which also found no associations with Family or Significant Others with MD [34, 36]. The same applied to Tylka et al. [27], who also did not find a significant association between romantic partners and Muscularity internalization or Muscularity dissatisfaction. Instead, partner pressure to be mesomorphic was associated with disordered eating behaviors. The authors explained this by the contextual nature of partner influence: men likely eat often with their romantic partners and may therefore restrict their eating when their partner is critical of their appearance or emphasizes muscularity and leanness. Regarding the lack of family influence in our sample, a study on male undergraduates by Galioto e al. [59] found that while both family and peer influence were correlated with muscularity-oriented body dissatisfaction, only peer influence remained significant when controlling for other social influences, suggesting that peer influence may be of greater relevance than family influence. In another qualitative study, participants also did not perceive their parents as enforcing stereotypical masculine standards [58]. However, this study included only a small sample of men, limiting generalizability. Differences in findings may also be attributable to different operationalizations and measurement of constructs, thus warranting further investigation. In contrast, eating disorders seem to be affected by a broader range of sociocultural influences, whereas in MD, peers and media seem to play a prominent role. Beyond the empirically documented impact of peer and media influences on eating disorders [33, 60–62], family-related factors, such as critical maternal comments and adverse family interactions [63, 64], as well as partnership-related conflicts and instability [65, 66] have been associated with eating disorder pathology. This pattern suggests that the sociocultural etiology of eating disorders may be more multifaceted and relationally embedded than that of MD, meriting further research.

When examining gender-specific mediation pathways, we observed some differences in how the various sociocultural pressures and forms of internalization contributed to MD. For men, Peer pressure also operated exclusively through Muscularity internalization, consistent with the total model. Media influenced MD via Muscularity and General Attractiveness internalization, but not via Thin/Low Body fat internalization, suggesting a less prominent role of the thin ideal and highlighting Muscularity internalization as a key mechanism in the development of MD, especially in men´s samples. In a study on eating disorders [67], both Peer and Media pressure also emerged as the strongest predictors for disordered eating in men, when operating via Muscular and Thin/Low Body fat internalization, while Family pressure and pressure from Significant Others did not play a role. However, only thinness showed a direct effect on disordered eating, while muscularity was mediated by appearance comparison, highlighting potentially differing pathways between MD and eating disorder-related processes in men. Future research is needed to further investigate this topic and to clarify the potentially differing underlying mechanisms. Surprisingly, for women, the only significant mediation pathway was from Family pressure via Muscularity internalization to MD, once again underscoring Muscularity internalization as the central mechanism. Thus, although Family did not play a role when examining the total sample, it seems to be of greater importance when focusing only on women. Since most studies on this topic are mostly focused on male populations, there are few studies examining pressure sources and internalization in the development of MD in women. A study by Mullick et al. [34] found the same associations of pressure sources and MD in women as in the total sample, while another study examining the effect of family pressure and athletic internalization on body image in women could not find a significant mediation [68], contrasting our findings. However, another study [69] showed that family influence may be stronger for women than for men, partially explaining the effect of Family in our sample; this study by Rodgers et al. showed that parental variables (such as maternal or paternal comments, pressure, or modeling) explained a larger amount of the variance in girls’ body dissatisfaction compared to boys [69], indicating a potentially greater importance of familial influences in women. This also aligns with the fact that eating disorders, which are generally more prevalent in women than in men [70], are strongly influenced by familial factors [63, 64]. This gender difference in influential factors may also apply to MD: similar mechanisms may contribute to Muscularity internalization, which in turn may increase the risk of developing MD in women. Overall, family-related factors may be more salient in shaping body-related attitudes in women. However, this association needs further investigation. It is also worth considering that, although participants flagged as having an eating disorder by the MD algorithm were excluded from the analyses, subclinical eating disorder symptoms may still have influenced the observed associations. This is particularly relevant for women, given the higher prevalence of such symptoms in this population. Because thinness orientation is itself a core eating disorder-relevant construct, the generally higher Thin/Low Body Fat internalization scores in women may partly reflect subclinical eating disorder pathology rather than MD-specific processes, which could in turn have shaped the estimates for this internalization domain. By the same logic, the family-related pathway observed in women -disordered eating itself being strongly influenced by familial factors- may partly capture broader body image- or eating disorder-relevant susceptibility rather than a mechanism specific to MD. The female-specific associations should therefore be interpreted with caution, as the present design cannot fully disentangle MD-specific from eating disorder-related variance.

Further, it is possible that, in addition to gender, gender roles play a role in Muscularity internalization. Brandt et al. [71] found that masculinity is associated with stronger muscularity ideals, whereas femininity shows weaker associations in women. Therefore, gender roles may also be relevant when examining family influences. Nevertheless, additional research is needed to better understand these relationships.

While gender roles provide important insight into sociocultural mechanisms of muscularity internalization and MD, an additional and increasingly relevant perspective considers differences across gender identities. MD symptoms and mechanisms may present differently in gender diverse individuals, such as transgender or gender-expansive people (i.e., those who identify outside of the man vs. woman binary), among other reasons due to the added influence of minority stress (e.g., discrimination, misgendering, and internalized transhostility), perceived gender-incongruence, and the pressure to be socially recognized as the desired gender [72–75]. For instance, MD symptoms have been found to be highest in transgender men, followed by gender-expansive people and transgender women [73]. Also, the impact of internalized body ideals, especially the athletic ideal, on MD symptoms (assessed via the MDDI) has been found to be dependent on gender identity and was especially strong for transgender men [75]. Further research regarding gender diverse individuals is warranted.

Importantly, all gender-specific analyses were exploratory and should be interpreted with caution, as the sample size of participants with MD is small when men and women are examined separately, potentially leading to unstable model estimates. Further research is warranted to examine gender-specific mediation pathways in the development of MD, especially in women and gender diverse populations. In addition, future studies should further investigate both shared and distinct sociocultural and internalization pathways underlying MD and eating disorder-related outcomes to better differentiate their underlying mechanisms. Taken together, the central role of Muscularity internalization in the development of MD has important implications for interventions. Rather than modifying exposure to sociocultural pressures, interventions should target the internalization of body ideals. In the case of MD, the Muscularity ideal should be addressed as the key mechanism in both genders, while internalized General Attractiveness may warrant additional attention specifically in men. These findings highlight the importance of tailoring preventive and therapeutic strategies to address the most relevant internal processes, which may differ between women and men. Additionally, these findings may also be relevant for eating disorder treatment and research, suggesting that interventions targeting internalization processes may differ across body image disorders, with muscularity internalization as a central target in MD and thin-ideal internalization in eating disorder-related outcomes. However, further research is necessary to examine shared and distinct pathways across both disorders.

### 4.1. Limitations

Several limitations must be noted. The study’s cross-sectional design does not allow for conclusions regarding causality. Also, all data were obtained via self-report, which may be influenced by biases such as social desirability or inaccuracies in recall. Another potential limitation that needs to be considered is the use of the algorithm to determine algorithm-defined MD; although it provides a differentiated approach to operationalizing clinically algorithm-defined MD, it does not replace a clinical diagnosis made by a trained professional. Further, the sample was weighted by age and gender to enhance generalizability; however, the initial overrepresentation of younger participants led to higher weights for older individuals, which may have affected the stability of estimates for older participants. It should also be noted that 23 participants reporting “other” gender identity or giving no information on their gender at all were excluded from the analyses due to the small subgroup size, thus limiting generalizability of the findings and highlighting the need for research with larger, more gender-diverse samples. In addition, cisgender and transgender status was not assessed among participants identifying as men or women; the male and female groups may therefore include transgender individuals, whose MD mechanisms may differ. Cautious interpretation is also warranted for gender-specific models, especially the one for women. Due to the small sample size, conclusions should be drawn carefully and regarded as exploratory. Despite these limitations, the study provides data on a large sample of the German general population providing new insights in the associations of sociocultural pressures, internalization of beauty standards, and MD.

### 4.2. Summary, conclusions and implications

This study examined the relationships between sociocultural pressures, internalization of beauty ideals, and algorithm-defined MD. Across the total sample, internalization of the Thinness, Muscularity, and General attractiveness ideals was significantly associated with algorithm-defined MD. Mediation analyses indicated that Media pressures influenced MD indirectly through all forms of internalization, reflecting their broad and pervasive impact. In contrast, peer pressure affected MD only via the Muscularity ideal. Overall, Muscularity internalization emerged as the strongest influence and the central mediating mechanism linking sociocultural pressures to MD. Gender-specific analyses revealed distinct patterns: For men, Muscularity internalization remained the central mediator of peer pressure on MD, with Media additionally influencing General attractiveness internalization. For women, Family pressure uniquely contributed to MD via Muscularity internalization, suggesting that familial influences may play a more substantial role for women than men in the internalization of Muscularity ideals. In summary, these findings align with the core premise of the Tripartite Influence Model, suggesting that sociocultural pressures primarily affect MD through the internalization of appearance ideals. The importance of internalization highlights the need for interventions targeting these internal processes, rather than focusing solely on reducing or modifying exposure to sociocultural pressures. In the case of MD, interventions should focus on the Muscularity ideal as the key mechanism in both genders; additionally, internalized General Attractiveness should be targeted specifically in men.

## Supplementary Information

Supplementary Material 1.

Supplementary Material 2.

Supplementary Material 3.

Supplementary Material 4.

Supplementary Material 5.

## Data Availability

Data are available upon reasonable request from the corresponding author.
