# Supplementary material for: The impact of peer and media pressure on muscle dysmorphia for men and women in the general German population is mediated by the internalization of muscularity ideals
Source: J Eat Disord. 2026 Aug 4;14:176. doi: 10.1186/s40337-026-01718-3 (PMC13435413; doi:10.1186/s40337-026-01718-3)
Supplement: Supplementary file 1 [file 40337_2026_1718_MOESM1_ESM.docx]

| **Supplementary Table S1.**  Muscle dysmorphia (MD) diagnostic criteria^1^ and operationalization in the current study^2^ | |
| --- | --- |
| Criteria | Operationalization |
| A. The person has a preoccupation with the idea that one’s body is not sufficiently lean and muscular. Characteristic associated behaviors include long hours of lifting weights and excessive attention to diet | ≥ 1.5 SD above the (gender-specific) mean on the DFS subscale of the MDDI^3^  To enhance the validity of Mitchison et al.'s (2021) original operationalization, the current study defined a participant's preoccupation with muscularity as having a score of ≥ 1.5 standard deviations above the mean on the Drive for Size subscale of the MDDI |
| B. The preoccupation causes clinically significant distress or impairment in social, occupational, or other important areas of functioning, as demonstrated by at least two of the following four criteria: |  |
| (a) The individual frequently gives up important social, occupational, or recreational activities because of a compulsive need to maintain his or her workout and diet schedule | A score of ≥4 (indicating often to always) on one or more of the following DMS/MDDI items:  1. “I think that my weight-training schedule interferes with other aspects of my  life” 2. “I pass up social activities with friends because of my workout schedule” 3. “I pass up chances to meet new people because of my workout schedule” |
| (b) The individual avoids situations where his or her body is exposed to others, or endures such situations only with marked distress or intense anxiety | A score of ≥ 4 (indicating moderately to markedly) on the EDE-Q item:  “Over the past 4 weeks (28 days), how uncomfortable have you felt about others seeing your shape or figure (for example, in communal changing rooms, when  swimming, or wearing tight clothes)?” |
| (c) The preoccupation about the inadequacy of body size or musculature causes clinically significant distress or impairment in social, occupational, or other important areas of functioning; | K10 total score ≥ 15 (indicating mild to severe distress) |
| (d) The individual continues to work out, diet, or use ergogenic (performance enhancing) substances despite knowledge of adverse physical or psychological consequences | Score ≥ 4 (indicating often to always) on the DMS item: “I lift weights to build up muscle” OR  Score ≥ 12 days on the researcher-developed item: “In the past 28 days, how many days have you been on a very high-protein diet as a means of controlling your shape or weight?” OR  Score ≥ 1 on the researcher-developed item: “In the past 28 days, how many times have you taken anabolic steroids as a means of increasing your muscularity?” |
| C. The primary focus of the preoccupation and behaviors is on being too small or inadequately muscular, as distinguished from fear of being fat, as in anorexia nervosa, or a primary preoccupation only with other aspects of appearance, as in other forms of BDD | Score ≥ 4 (indicating often to always) on the DMS item: “I wish that I were more muscular”  AND  Exclusion, if meeting criteria for anorexia nervosa, bulimia nervosa, atypical anorexia nervosa, or subthreshold bulimia nervosa^4^ |

SD = Standard deviation; DFS = Drive for Size; MDDI = Muscle Dysmorphic Disorder Inventory; DMS = Drive for Muscularity Scale; EDE-Q = Eating Disorder Examination Questionnaire; K10 = Kessler 10; BDD = Body dysmorphic disorder

^1^ Based on Pope, H. G., Gruber, A. J., Choi, P., Olivardia, R., & Phillips, K. A. (1997). Muscle dysmorphia: An underrecognized form of body dysmorphic disorder. *Psychosomatics*, *38*(6), 548–557. <https://doi.org/10.1016/S0033-3182(97)71400-2>

^2^ Adapted from Mitchison, D., Mond, J., Griffiths, S., Hay, P., Nagata, J. M., Bussey, K., … Murray, S. B. (2022). Prevalence of muscle dysmorphia in adolescents: findings from the EveryBODY study. *Psychological Medicine*, *52*(14), 3142–3149. doi:10.1017/S0033291720005206

^3^ Only minor change to Mitchison et al.’s operationalization introduced by Ganson KT, Testa A, Rodgers RF, Murray SB, Nagata JM. Exploring body ideal internalization, sociocultural pressures, and probable muscle dysmorphia in Canadian and American boys and men. Eat Behav. 2025 Jan;56:101944. doi: 10.1016/j.eatbeh.2025.101944. Epub 2025 Jan 10. PMID: 39799703.FormularbeginnFormularende

^4^ For eating disorder criteria, see Mitchison, D., Mond, J., Bussey, K., Griffiths, S., Trompeter, N., Lonergan, A., … Hay, P. (2020). DSM-5 full syndrome, other specified, and unspecified eating disorders in Australian adolescents: prevalence and clinical significance. *Psychological Medicine*, *50*(6), 981–990. doi:10.1017/S0033291719000898. Operationalization not shown.
