# Supplementary material for: The impact of peer and media pressure on muscle dysmorphia for men and women in the general German population is mediated by the internalization of muscularity ideals
Source: J Eat Disord. 2026 Aug 4;14:176. doi: 10.1186/s40337-026-01718-3 (PMC13435413; doi:10.1186/s40337-026-01718-3)
Supplement: Supplementary file 2 [file 40337_2026_1718_MOESM2_ESM.docx]

Supplementary Table S2: Description of questionnaires used for MD operationalization and assesssment

#### The Muscle Dysmorphic Disorder Inventory

The Muscle Dysmorphic Disorder Inventory (MDDI) (1) is a multidimensional assessment tool measuring MD comprising three factors, such as “Drive for Size” (the desire to increase muscularity and strength), “Appearance intolerance” (negative beliefs about one’s body appearance), and “functional impairment” (interference with daily functioning). 13 Items are rated on a 5-Point-Likert Scale ranging from “Never” to “Always”. The German version (2) uses a Cut-Off of >39 to classify individuals as “at risk” for MD. It demonstrated good psychometric properties, including acceptable internal consistency, meaningful associations with related constructs, and a stable factor structure across genders.

#### The Drive for Muscularity Scale

The Drive for Muscularity Scale (DMS) (3) is a 15-item self-report questionnaire designed to assess the pursuit for muscularity. Every item is rated on a 6 Point-Likert-type scale from “Always” to “Never”. The DMS consists of two subscales: “muscularity-related attitudes”, which assess dissatisfaction with musculartiy, and “muscle-enhancing behaviors”, which assesses engaging in muscle-building behaviors. Evidence also supports the use of a global factor representing overall drive for muscularity. Equal to the English original, the German version (4) demonstrated good internal consistency and validity.

#### The Kessler Psychological Distress Scale

The Kessler Psychological Distress Scale (K10) (5) is a 10-item self-report questionnaire to assess general psychological distress in the past 30 days. It measures the frequency of several anxiety and depressive symptoms on a 5-point Likert scale ranging from *“None of the time”* to *“All of the time.”* Total scores range from 10 to 50, with higher scores indicating greater levels of distress. Scores above 15 suggest mild distress, whereas scores above 30 indicate severe distress. The German version of the K10 scale (6) exhibits psychometric properties comparable to those of the original English version. It demonstrated good internal consistency, satisfactory correlations with conceptually related scales, and very good discriminative ability.

#### Eating Disorder Examination-Questionnaire

The Eating Disorder Examination-Questionnaire (EDE-Q) (7) has a total of 28 items, assessing the range, frequency, and severity of behaviors as well as attitudes associated with eating disorder psychopathology over the past 28 days. It comprises four subscales: Restraint, Eating Concern, Weight Concern, and Shape Concern, as well as a global score. For this study, we used the German version of the EDE-Q (8), which demonstrated high internal consistency, temporal stability, and good convergent validity. It effectively discriminated between clinical and nonclinical groups and detected changes following intervention.

1.	Hildebrandt T, Langenbucher J, Schlundt DG. Muscularity concerns among men: Development of attitudinal and perceptual measures. Body Image. 2004;1(2):169–81.

2.	Zeeck A, Welter V, Alatas H, Hildebrandt T, Lahmann C, Hartmann A. Muscle Dysmorphic Disorder Inventory (MDDI): Validation of a German version with a focus on gender. PLoS One. 2018;13(11):1–13.

3.	McCreary DR. The Drive for Muscularity Scale: Description, Psychometrics, and Research Findings. The muscular ideal: Psychological, social, and medical perspectives. Washington, DC, US: American Psychological Association; 2007. p. 87–106.

4.	Waldorf M, Cordes M, Vocks S, McCreary D. „Ich wünschte, ich wäre muskulöser”: Eine teststatistische Überprüfung der deutschsprachigen Fassung der Drive for Muscularity Scale (DMS). Diagnostica. 2014;

5.	Kessler RC, Andrews G, Colpe LJ, Hiripi E, Mroczek DK, Normand SLT, et al. Short screening scales to monitor population prevalences and trends in non-specific psychological distress. Psychol Med. 2002;32:959–76.

6.	Giesinger J, Rumpold G, Schüßler G. Die K10-Screening-Skala für unspezifischen psychischen Distress. Psychosom und Kons. 2008;2(2):103–10.

7.	Fairburn CG, Beglin SJ. Assessment of eating disorders: interview or self-report questionnaire? Int J Eat Disord. 1994 Dec;16(4):363–70.

8.	Hilbert A, Tuschen-Caffier B, Karwautz A, Niederhofer H, Munsch S. Eating Disorder Examination-Questionnaire. Diagnostica. 2007 Jul 1;53(3):144–54.
