# Supplementary material for: The impact of peer and media pressure on muscle dysmorphia for men and women in the general German population is mediated by the internalization of muscularity ideals
Source: J Eat Disord. 2026 Aug 4;14:176. doi: 10.1186/s40337-026-01718-3 (PMC13435413; doi:10.1186/s40337-026-01718-3)
Supplement: Supplementary file 3 [file 40337_2026_1718_MOESM3_ESM.docx]

Supplementary Tables S3a-c: Correlation matrices for SATAQ Scales

Supplementary Table S3a: Correlation Matrix Total Sample

|  | Internalization: Thin/Low Body Fat | Internalization: Muscular | Internalization: General Attractiveness | Pressure: Family | Pressure: Peers | Pressure: Significant Others | Pressure: Media |
| --- | --- | --- | --- | --- | --- | --- | --- |
| Internalization: Thin/Low Body Fat | 1 | .306 | .412 | .291 | .348 | .360 | .465 |
| Internalization: Muscular | .306 | 1 | .378 | .114 | .312 | .201 | .216 |
| Internalization: General Attractiveness | .412 | .378 | 1 | .139 | .230 | .190 | .342 |
| Pressure: Family | .291 | .114 | .139 | 1 | .532 | .637 | .459 |
| Pressure:  Peers | .348 | .312 | .230 | .532 | 1 | .713 | .553 |
| Pressure: Significant Others | .360 | .201 | .190 | .637 | .713 | 1 | .566 |
| Pressure: Media | .465 | .216 | .342 | .459 | .553 | .566 | 1 |

Supplementary Table S3b: Correlation Matrix Men

|  | Internalization: Thin/Low Body Fat | Internalization: Muscular | Internalization: General Attractiveness | Pressure: Family | Pressure: Peers | Pressure: Significant Others | Pressure: Media |
| --- | --- | --- | --- | --- | --- | --- | --- |
| Internalization: Thin/Low Body Fat | 1 | .386 | .244 | .258 | .338 | .305 | .366 |
| Internalization: Muscular | .386 | 1 | .376 | .172 | .441 | .303 | .385 |
| Internalization: General Attractiveness | .244 | .376 | 1 | .180 | .299 | .227 | .307 |
| Pressure: Family | .258 | .172 | .180 | 1 | .435 | .563 | .399 |
| Pressure:  Peers | .338 | .441 | .299 | .435 | 1 | .696 | .615 |
| Pressure: Significant Others | .305 | .303 | .227 | .563 | .696 | 1 | .642 |
| Pressure: Media | .366 | .385 | .307 | .399 | .615 | .642 | 1 |

Supplementary Table S3c: Correlation Matrix Women

|  | Internalization: Thin/Low Body Fat | Internalization: Muscular | Internalization: General Attractiveness | Pressure: Family | Pressure: Peers | Pressure: Significant Others | Pressure: Media |
| --- | --- | --- | --- | --- | --- | --- | --- |
| Internalization: Thin/Low Body Fat | 1 | .265 | .603 | .306 | .377 | .415 | .534 |
| Internalization: Muscular | .265 | 1 | .499 | .121 | .144 | .090 | .140 |
| Internalization: General Attractiveness | .603 | .499 | 1 | .069 | .198 | .175 | .342 |
| Pressure: Family | .306 | .121 | .069 | 1 | .649 | .712 | .477 |
| Pressure:  Peers | .377 | .144 | .198 | .649 | 1 | .730 | .557 |
| Pressure: Significant Others | .415 | .090 | .175 | .712 | .730 | 1 | .539 |
| Pressure: Media | .534 | .140 | .342 | .477 | .557 | .539 | 1 |
