# Supplementary material for: The impact of peer and media pressure on muscle dysmorphia for men and women in the general German population is mediated by the internalization of muscularity ideals
Source: J Eat Disord. 2026 Aug 4;14:176. doi: 10.1186/s40337-026-01718-3 (PMC13435413; doi:10.1186/s40337-026-01718-3)
Supplement: Supplementary file 4 [file 40337_2026_1718_MOESM4_ESM.docx]

Supplementary Tables S4a-f

*Supplementary Table S4a. Binary logistic regression model: SATAQ Scales as Predictors of Algorithm-defined MD (X🡪Y) in men*

| DV: MD | B | SE | OR (Exp [B]) | 95% CI for OR | Sig. |
| --- | --- | --- | --- | --- | --- |
| Age | -0.027 | 0.019 | 0.973 | 0.937 - 1.010 | 0.152 |
| BMI | -0.246 | 0.060 | 0.782 | 0.695 - 0.880 | < .001* |
| Thin/Low Body Fat | -0.324 | 0.238 | 0.724 | 0.454 - 1.154 | 0.174 |
| Muscular | 1.375 | 0.382 | 3.957 | 1.867 - 8.383 | < .001* |
| General Attractiveness | 0.715 | 0.219  0.313 | 2.044 | 1.331 - 3.140 | < .001* |
| Family | -0.091 | 0.313 | 0.912 | 0.494 - 1.689 | 0.772 |
| Peers | -0.058 | 0.230 | 0.944 | 0.600 - 1.484 | 0.802 |
| Significant Others | -0.326 | 0.322 | 0.722 | 0.383 - 1.358 | 0.311 |
| Media | 0.414 | 0.224 | 1.513 | 0.975 - 2.349 | 0.065 |

Note: *Results differed significantly on a p<.05 level.

Supplementary Table S4b. Linear regression models: Effects of sociocultural pressure sources on internalization (X🡪M) in men

| DV: Thin/Low Body Fat | B | SE | 95% CI for B | Sig. |
| --- | --- | --- | --- | --- |
| Age | 0.011 | 0.008 | -0.003 - 0.026 | 0.133 |
| BMI | -0.007 | 0.014  0.125 | -0.035 0.021  -0.113 0.379 | 0.639 |
| Family | 0.133 | 0.125 | -0.113 0.379  0.054 0.440 | 0.289 |
| Peers | 0.247 | 0.098 | 0.054 0.440 | 0.012* |
| Significant Others | -0.042 | 0.122 | -0.282 0.198 | 0.732 |
| Media | 0.254 | 0.067 | 0.123 0.385 | <0.001 |
| DV: Muscular |  |  |  |  |
| Age | -0.007  -0.038 | 0.007 | -0.021 - 0.007 | 0.299 |
| BMI | -0.038  0.026 | 0.014 | -0.066 - -0.011 | 0.006* |
| Family | 0.026  0.389 | 0.103 | -0.177 - 0.229 | 0.802 |
| Peers | 0.389 | 0.109 | 0.175 - 0.603 | <0.001* |
| Significant Others | -0.073 | 0.110 | -0.288 - 0.142 | 0.507 |
| Media | 0.180 | 0.066 | 0.051 - 0.309 | 0.006* |
| DV: General Attractiveness |  |  |  |  |
| Age | -0.006 | 0.007 | -0.019 0.008  -0.013 0.051 | 0.416 |
| BMI | 0.019 | 0.016 | -0.013 0.051 | 0.243 |
| Family | 0.058 | 0.156 | -0.247 0.364 | 0.708 |
| Peers | 0.224 | 0.109 | 0.011 0.437 | 0.039* |
| Significant Others | -0.104 | 0.155 | -0.408 0.200 | 0.502 |
| Media | 0.192 | 0.077 | 0.040 0.343 | 0.013* |

Note: *Results differed significantly on a p<.05 level.

Supplementary Table S4c. Indirect paths of the mediation model (X🡪M🡪Y) in men

| X🡪M🡪Y | Indirect effect (ab) | 95% CI ab | Sig. |
| --- | --- | --- | --- |
| Family → Thin → MD | -0.04 | -0.144 to 0.058 | p = .403 |
| Peers → Thin → MD | -0.08 | -0.211 to 0.051 | p = .232 |
| SO → Thin → MD | 0.01 | -0.066 to 0.094 | p = .740 |
| Media → Thin → MD | -0.08 | -0.208 to 0.044 | p = .200 |
| Family → Musc → MD | 0.04 | -0.243 to 0.315 | p = .802 |
| Peers → Musc → MD | 0.54 | 0.121 to 0.949 | p = .011* |
| SO → Musc → MD | -0.10 | -0.401 to 0.200 | p = .514 |
| Media → Musc → MD | 0.25 | 0.024 to 0.470 | p = .030* |
| Family → GA → MD | 0.04 | -0.178 to 0.261 | p = .710 |
| Peers → GA → MD | 0.16 | -0.020 to 0.340 | p = .081 |
| SO → GA → MD | -0.07 | -0.296 to 0.147 | p = .510 |
| Media → GA → MD | 0.14 | 0.001 to 0.273 | p = .048* |

Note: *Results differed significantly on a p<.05 level. BMI and age were included as control variables.

*Supplementary Table S4d. Binary logistic regression model: SATAQ Scales as Predictors of Algorithm-defined MD (X🡪Y) in women*

| DV: MD | B | SE | OR (Exp [B]) | 95% CI for OR | Sig. |
| --- | --- | --- | --- | --- | --- |
| Age | -0.045 | 0.023 | 0.956 | 0.914 - 1.001 | 0.055 |
| BMI | -0.078 | 0.113 | 0.925 | 0.740 - 1.155 | 0.489 |
| Thin/Low Body Fat | -0.769 | 0.395 | 0.464 | 0.214 - 1.006  2.188 22.546 | 0.055 |
| Muscular | 1.949 | 0.594 | 7.024 | 2.188 - 22.546 | 0.001*  0.221 |
| General Attractiveness | 0.733 | 0.598 | 2.081 | 0.642 - 6.738 | 0.221  0.428 |
| Family | 0.346 | 0.437 | 1.414 | 0.599 - 3.337 | 0.428 |
| Peers | 1.360 | 0.758 | 3.895 | 0.879 - 17.259 | 0.073 |
| Significant Others | -0.233 | 0.444 | 0.792 | 0.331 - 1.894 | 0.600  0.081 |
| Media | -0.918 | 0.526 | 0.399 | 0.142 - 1.121 | 0.081 |

Note: *Results differed significantly on a p<.05 level.

Supplementary Table S4e. Linear regression models: Effects of sociocultural pressure sources on internalization (X🡪M) in women

| DV: Thin/Low Body Fat | B | SE | 95% CI for B | Sig. |
| --- | --- | --- | --- | --- |
| Age | -0.015 | 0.004 | -0.023 - -0.008 | <0.001* |
| BMI | -0.045 | 0.011 | -0.066 - -0.023 | <0.001* |
| Family | 0.063 | 0.067 | -0.069 - 0.194 | 0.348 |
| Peers | -0.035 | 0.089 | -0.209 - 0.139 | 0.689 |
| Significant Others | 0.255 | 0.092 | 0.075 - 0.435 | 0.006* |
| Media | 0.278 | 0.056 | 0.168 - 0.389 | <0.001* |
| DV: Muscular |  |  |  |  |
| Age | 0.001 | 0.006 | -0.010 - 0.012 | 0.836 |
| BMI | -0.063 | 0.015 | -0.092 - -0.033 | <0.001* |
| Family | 0.230 | 0.074 | 0.085 - 0.374 | 0.002* |
| Peers | 0.093 | 0.103 | -0.109 - 0.295 | 0.366 |
| Significant Others | -0.106 | 0.114 | -0.329 - 0.117 | 0.350 |
| Media | 0.079 | 0.062 | -0.043 - 0.201 | 0.203 |
| DV: General Attractiveness |  |  |  |  |
| Age | -0.008 | 0.005 | -0.018 - 0.001 | 0.094 |
| BMI | -0.060 | 0.013 | -0.086 - -0.034 | <0.001* |
| Family | 0.001 | 0.058 | -0.112 - 0.115 | 0.980 |
| Peers | 0.023 | 0.057 | -0.089 - 0.135 | 0.684 |
| Significant Others | 0.057 | 0.077 | -0.094 - 0.208 | 0.458 |
| Media | 0.184 | 0.043 | 0.100 - 0.269 | <0.001* |

Note: *Results differed significantly on a p<.05 level.

Supplementary Table S4f. Indirect paths of the mediation model (X🡪M🡪Y) in women

| X🡪M🡪Y | Indirect effect (ab) | 95% CI ab | Sig. |
| --- | --- | --- | --- |
| Family → Thin → MD | -0.05 | -0.160 to 0.064 | p = .398 |
| Peers → Thin → MD | 0.03 | -0.109 to 0.164 | p = .695 |
| SO → Thin → MD | -0.20 | -0.437 to 0.045 | p = .111 |
| Media → Thin → MD | -0.21 | -0.446 to 0.017 | p = .070 |
| Family → Musc → MD | 0.45 | 0.059 to 0.836 | p = .024* |
| Peers → Musc → MD | 0.18 | -0.226 to 0.589 | p = .383 |
| SO → Musc → MD | -0.21 | -0.658 to 0.244 | p = .369 |
| Media → Musc → MD | 0.15 | -0.100 to 0.410 | p = .235 |
| Family → GA → MD | 0.00 | -0.082 to 0.084 | p = .980 |
| Peers → GA → MD | 0.02 | -0.069 to 0.103 | p = .699 |
| SO → GA → MD | 0.04 | -0.087 to 0.171 | p = .526 |
| Media → GA → MD | 0.13 | -0.090 to 0.360 | p = .239 |

Note: *Results differed significantly on a p<.05 level. BMI and age were included as control variables.
