# Supplementary material for: The impact of peer and media pressure on muscle dysmorphia for men and women in the general German population is mediated by the internalization of muscularity ideals
Source: J Eat Disord. 2026 Aug 4;14:176. doi: 10.1186/s40337-026-01718-3 (PMC13435413; doi:10.1186/s40337-026-01718-3)
Supplement: Supplementary file 5 [file 40337_2026_1718_MOESM5_ESM.docx]

Supplementary Figures S1-2

*Figure S1. Significant indirect Paths in the Mediation Model for men*

Figure S2. Significant indirect Paths in the Mediation Model for women
